# Federated Compressed Learning Edge Computing Framework with Ensuring Data Privacy for PM2.5 Prediction in Smart City Sensing Applications

**DOI:** 10.3390/s21134586

**Published:** 2021-07-04

**Authors:** Karisma Trinanda Putra, Hsing-Chung Chen, Prayitno, Marek R. Ogiela, Chao-Lung Chou, Chien-Erh Weng, Zon-Yin Shae

**Affiliations:** 1 Department of Computer Science and Information Engineering, Asia University, Taichung City 413, Taiwan; karisma@ft.umy.ac.id (K.T.P.); prayitno@polines.ac.id (P.); zshae1@asia.edu.tw (Z.-Y.S.); 2 Department of Electrical Engineering, Universitas Muhammadiyah Yogyakarta, Bantul 55183, Indonesia; 3 Department of Medical Research, China Medical University Hospital, China Medical University, Taichung City 404, Taiwan; 4 Department of Electrical Engineering, Politeknik Negeri Semarang, Semarang 50275, Indonesia; 5 Cryptography and Cognitive Informatics Laboratory, AGH University of Science and Technology, 30-059 Krakow, Poland; mogiela@agh.edu.pl; 6 Department of Computer Science and Information Engineering, Chung Cheng Institute of Technology, National Defense University, Taoyuan City 334, Taiwan; chaolung.chou@gmail.com; 7 Department of Electrical Communication Engineering, National Kaohsiung University of Science and Technology, Kaohsiung City 811, Taiwan; ceweng@nkust.edu.tw

**Keywords:** federated compressed learning, data privacy, smart city sensing

## Abstract

The sparse data in PM2.5 air quality monitoring systems is frequently happened on large-scale smart city sensing applications, which is collected via massive sensors. Moreover, it could be affected by inefficient node deployment, insufficient communication, and fragmented records, which is the main challenge of the high-resolution prediction system. In addition, data privacy in the existing centralized air quality prediction system cannot be ensured because the data which are mined from end sensory nodes constantly exposed to the network. Therefore, this paper proposes a novel edge computing framework, named Federated Compressed Learning (FCL), which provides efficient data generation while ensuring data privacy for PM2.5 predictions in the application of smart city sensing. The proposed scheme inherits the basic ideas of the compression technique, regional joint learning, and considers a secure data exchange. Thus, it could reduce the data quantity while preserving data privacy. This study would like to develop a green energy-based wireless sensing network system by using FCL edge computing framework. It is also one of key technologies of software and hardware co-design for reconfigurable and customized sensing devices application. Consequently, the prototypes are developed in order to validate the performances of the proposed framework. The results show that the data consumption is reduced by more than 95% with an error rate below 5%. Finally, the prediction results based on the FCL will generate slightly lower accuracy compared with centralized training. However, the data could be heavily compacted and securely transmitted in WSNs.

## 1. Introduction

With the increasing air pollution in recent years, especially in industrialized countries, toxic substances attached to the particulate matter have entered the human body through the respiratory system. The Health Effects Institute (HEI) reported in 2018 that the over 95 percent of the world’s population is breathing unhealthy air. In 2018, long-term exposure to air pollution contributes to the deaths of 6.1 million people with strokes, heart attacks, lung disease, and lung cancer [1]. One of the air pollutants is particulate matter (PM) with a size below 2.5 μm (PM2.5). These micro particles are the most dangerous forms of air pollution because they can penetrate deep into the lungs [2]. For every 10 μg/m increase in PM pollutants, the risk of lung cancer increases by 22% [3]. To combat urban air pollution, scientists need long-term efforts in collecting pollution data to build an accurate air pollution prediction system. The effort includes the implementation of the ideal smart city. The concept of the ideal smart city allows microelectronic devices to be installed in urban areas to collect environmental pollution data accurately. This concept needs sensory nodes that are spread massively, evenly, and integrated into the sensor network system. However, the sparse data and missing records are always found in this extensive network [4,5,6]. These problems appear due to the massive node deployment with uneven and inefficient distribution, the data losses during the transmission process, and the failure nodes. The accuracy of the prediction system decreases due to duplicated, corrupted, and unbalanced data in the data mining process. Therefore, the massive sparse sensory data affected by inefficient node deployment, insufficiency of communication, and fragmented records become the main challenge of a high-resolution prediction system.

Recently, more and more scientists have started to utilize edge computing technology to solve the main challenge [7,8,9] mentioned above. The edge computing technology allows centralized computing to be distributed to smaller nodes on the edge of networks [10]. This technology can be implemented in air quality prediction systems that perform measurements over thousands or even millions of sensory nodes. The implementation is realized by using micro-sensor nodes, e.g., Internet of Things (IoT) nodes, which are connected to cloud servers via the Internet. In addition, IoT technology opens up new possibilities, especially for the development of sensor nodes that are smart, energy-efficient, and easy to be implemented, although at the expense of higher data latency and smaller computing resources [11]. However, the more sensor variants and the denser the sampling rates, the more congested the data traffic, the higher the use of processing resources and the power consumption in a WSN. Compressed sensing (CS) techniques can generate an efficient amount of data while maintaining data integrity in the WSN [12,13,14]. Meanwhile, in an established prediction system (i.e., Airbox system), centralized servers record the user’s address, geolocation, timestamp, PM concentration, temperature, and humidity data to generate predictions and visualizations. A system owned by the government monitors urban air quality using many sensors, but with a lower resolution. On the other hand, with the support from the community, the existing system offers a more massive spread of sensory nodes with a higher sampling rate. However, with a higher sampling rate, the existing system generates a lot of data which sometimes does not contribute much to prediction accuracy and increases the possibility of serious data leaks, e.g., side-channel attack. There is a significant risk for the users related to privacy issues with the large number of nodes that record all activities and conditions at their residential areas [15,16]. The previous study has shown that a simple piece of information can be used as knowledge material for third parties, e.g., the microenvironmental concentration of PM2.5 is strongly influenced by user activities [17,18]. The current system is also prone to be misused by certain parties, such as during the mayoral election, where data representing the government’s performance becomes very valuable. The proposed scheme allows the public to be a cross-validator for the condition of their city without exposing their identity while educating residents to implement efforts to reduce air pollution levels. Therefore, efficient data generation and privacy issues are the main concerns in the development of smart city sensing.

To overcome these problems, a new framework named Federated Compressed Learning (FCL) is presented in this study to generate data efficiently while maintaining data privacy. By implementing compressed sensing, the networks’ data traffic can be significantly reduced. Meanwhile, federated learning ensures that raw data are not exposed to the network. In contrast to the centralized learning model, FCL inherits the basic ideas of compression technique, regional joint learning, and also considers the secure data exchange. FCL can leave the training data distributed on the secure fog coordinators and learn a shared model by aggregating compressed parameters locally. This proposed framework covers system architecture from end devices to cloud servers. At the low level, several sensor nodes record data, apply compression techniques, and send the compressed data to the coordinator. At the mid-level, several coordinators work together on an aggregated training scheme to produce a shared knowledge that could be used by the prediction model. Finally, at the top level, cloud servers (i.e., aggregation servers) manage the federated learning process and provide a prediction interface to the end users.

The novelties and contributions of this paper are summarized as follows. First, a novel framework based on Wi-Fi and ZigBee protocols is proposed to serve thousands of sensor nodes connected in smart cities. The ZigBee network is used at the lower network to cover more nodes in a broader area. Meanwhile, the Wi-Fi network is used to link the coordinators with the aggregation server. Second, end devices (i.e., sensor nodes) are required to transmit data continuously from a low-powered wireless network (i.e., ZigBee) to a high-powered wireless network (i.e., Wi-Fi) without sacrificing data error rates. A hybrid device that bridges these two communication protocols is designed. Third, the CS technique is implemented to reduce the data collected from the edge of the network. Meanwhile, data savings on the upper network are carried out by aggregating local model (i.e., a collection of weights) in FL scheme. Fourth, federated learning is implemented to reduce the network congestion while maintaining data privacy. Each small network under a coordinator node only sends its local model to the aggregation server. This aggregation scheme ensures significant information leakage (i.e., raw data) is not exposed on the networks. Finally, this study uses LSTM networks to generate predictions of PM2.5 concentrations. A comparative experiment is carried out to evaluate the performance of the scheme. In addition, the reconstructed and forecasted data is presented in the results.

The remainder of this paper is organized as follows. Section 2 provides an overview of the works related to the WSN-based prediction system in the centralized and decentralized scheme. In Section 3, the proposed prototypes are described in detail, including the data compression technique. Section 4 describes the edge computing scheme by using federated learning. Furthermore, evaluation scores and descriptions of the results are presented in Section 5. Section 6 provides discussion and, finally, a brief conclusion is presented in Section 7.

## 2. Related Works

### 2.1. Compressed Sensing

Recently, WSN has been developed into intelligent computing nodes that could be deployed massively by considering its scalability and power-saving capabilities. With the increasing number of nodes in WSNs, it is challenging to maintain the nodes that have been distributed in a large area over a long period of time. There is a possibility of performance degradation in several nodes or even crashes. Researchers used many approaches to make the WSN system easily implemented, especially in smart city sensing [19,20]. The sensor nodes with Wi-Fi capability are mostly used on WSN that are widely already available in urban areas. Although this technology is limited to its smaller coverage services, it can be quickly implemented as many devices already support this protocol. WSN can be expanded by using several protocols, e.g., Bluetooth, ZigBee, LoRa, NB-IoT, or even LTE networks. This study introduces a hybrid topology to expand the WSN service area by combining ZigBee-based sensory node and Wi-Fi-based edge computing technology. By using ZigBee networks, a larger number of nodes and a longer distance can be achieved better than by using Wi-Fi networks. A ZigBee network can serve clients up to 224 devices in a mesh configuration with a range of up to 100 m in urban area and up to 1 km outdoor. However, with the massive deployment of sensor nodes, there are possibilities that incomplete records and information leaks emerge due to the large amount of data being transmitted in the network. A compressed sensing technique can be implemented on WSNs to generate a small amount of data without sacrificing data fidelity [21,22]. Furthermore, the higher the data fidelity, the more accurate the performance of the AI model in predicting or making decisions [23].

Various CS techniques have been developed to achieve various WSN requirements, and many of them are considered lossy compression. As described in [22], the CS is a technology that utilizes fewer data (than those in the Nyquist–Shannon theorem [24]) to reconstruct the original data, as long as the data is compressible in particular transform theorem. The classical transform theorems (i.e., lossy compression) include Fourier transform, Hadamard transform, discrete cosine transform, and discrete wavelet transform are proven to be used to reduce communication overhead. Lossy compression can reduce data size significantly with a small error rate [25,26]. In some previous applications, the compression often shows small data changes even, quite sharp deformations appear in a small data area. In observation applications such as climate data analysis [27], a meaningful analysis can be investigated from the reconstructed data. The results reveal a small difference in average error rate using the compression rate of up to 80% of the original data. It can be seen from the reconstructed signal that is statistically indistinguishable from the original.

### 2.2. Privacy Issues in Smart City Sensing

Privacy is a big issue in smart city sensing because residents’ personal data is precious, and it will be more vulnerable with the increasing number of monitoring sites. These valuable data is associated with the personal information or location of resident. In a centralized system, a huge amount of raw data from end devices is collected by central servers [28] and protected by using trust-based service management protocol [29], e.g., IoT-HiTrust [30]. However, the system transfers data over the network, allowing the leakage of critical data [31] and the increasing risk of side-channel attack [32]. The edge computing framework utilizes federated learning technology, prevents direct access to the data, moves the compute resource to the edge, and prevents the raw data exchange to the central server [33,34,35,36]. For example, a smartphone that collects location data allows weather forecasting applications to directly access the user’s location, which violates information-based privacy. On the other hand, by using FL scheme, applications are only allowed to access the machine learning (ML) model without compromising data privacy. With this technology, every edge computing node in different areas contributes to the model training globally, while keeping the training data locally. In this study, every edge computing node (i.e., coordinator node) trains its local model by using a local dataset instead of uploading the dataset to central servers.

### 2.3. PM2.5 Prediction System

There is a risk to the population’s health because of industries’ development with their residual products that pollute the air [37]. Taiwan has installed 77 climate stations to monitor air pollutants. These stations are assisted by thousands of small sensor nodes installed throughout Taiwan to generate precise measurements. A study indicates that the rate of pollutant distribution varies depending on the season, wind direction, condition of the industrial area, and how wide the area is monitored [38]. A large number of data is generated during the data acquisition, and the temporal patterns appear in the process. To gather the temporal data that has a strong correlation, i.e., structurally related in some specific temporal moments, a large number of sensory nodes require to be installed with an identical distribution. An integrated sensor network is needed to measure data efficiently without sacrificing the data precision. Data with temporal characteristics present temporal dependencies, in which instances are not independent or identically distributed. It means that samples can be structurally related in some specific temporal moments. The instances change their class attribute depending on time. Thus, traditional prediction methods cannot be used in processing the data with temporal characteristics. These methods result in poor performance and misleading interpretation [39]. This study proposes a new framework to predict air quality related to PM2.5 by using a compressed dataset collected by coordinator nodes in a WSN. Each coordinator node receives compressed data from sensor nodes. Furthermore, a federated learning scheme is combined with compressed sensing to gather the data efficiently and securely. Federated learning ensures that the original data is not exposed from the outside network because only models are sent to the aggregation server. The neural network model (i.e., LSTM network) is utilized in this framework. LSTM has been shown to generate better prediction than other neural network models, especially on sequence data [40,41]. Finally, this study presents a system architecture for a massive-scale WSN that combines CS and FL in an FCL scheme to support smart city sensing.

## 3. Designing of Sensor Nodes Based on Compressed Sensing

**Figure 1:**
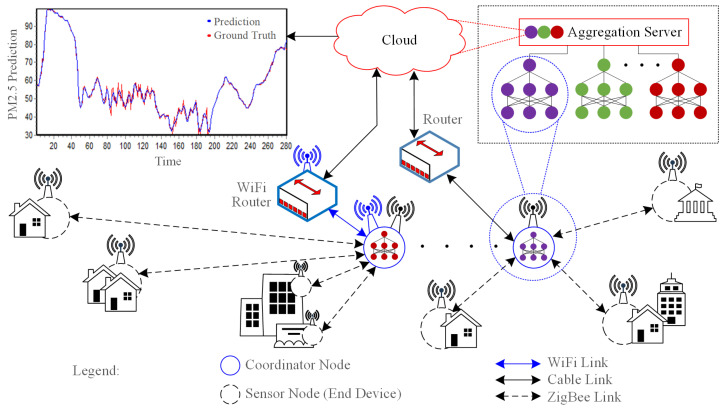
The proposed system architecture utilizes a hybrid combination of Zigbee and Wi-Fi network by considering local data collector serves by several coordinator nodes.

Wi-Fi-based sensory nodes are easily implemented in urban areas. However, these nodes are difficult to implement in suburban areas that are not covered by Wi-Fi signals. In fact, the source of air pollution not only comes from industrial areas and city traffics, but also from areas outside the city. The source of PM pollution also comes from carbon-burning smoke, especially during forest fires. The PM measurements over a wider area provide the PM propagation from time to time from its sources to residential areas. A new scheme to deploy the PM monitoring nodes is introduced in this section by considering the smart city concept. This scheme uses a combination of ZigBee and Wi-Fi network technology to increase its coverage area. The ZigBee protocol provides a low latency link that allows hundreds of nodes to be connected in a mesh network; thus, it can deal with data with a finer sampling rate. This protocol is easier to be implemented in cluster-tree WSNs and compatible with distributed computing schemes. The details of the proposed scheme are seen in Figure 1, where several sensor nodes are served by a coordinator node by using the ZigBee network. Several coordinators are connected to an aggregation server in the FL scheme via Wi-Fi network and they will contribute to the aggregated learning. This section describes the CS algorithm, hardware design, and pseudocode, which are afterwards implemented on sensor nodes.

### 3.1. Compression Algorithm

**Figure 2:**
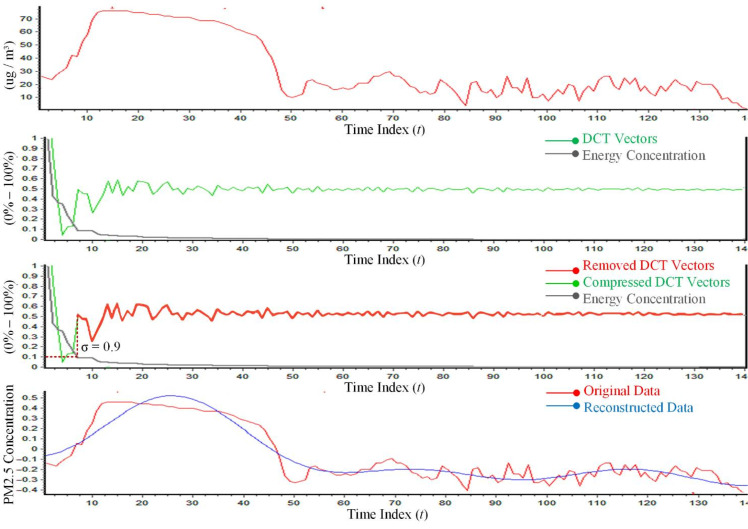
A sample of one-day measurement of PM2.5 collected and processed using DCT compression.

A signal in Discrete Cosine Transform (DCT) is represented as a sum of a sinusoid of varying magnitudes and frequencies. The DCT is one of the lossy compression techniques. This study uses DCT compression because it has very strong energy compaction properties [25,42]. As shown in Figure 2, by applying the DCT-II variant, more data will be stored in the lower frequency vectors. A large amount of information is compacted in a very low-frequency component of a signal and the rest (i.e., the higher frequency components) can be removed. This information is stored by using very few bits. The DCT vectors with a higher value than the energy concentration threshold, are saved because they significantly impact the data reconstruction.

The stages of the DCT compression algorithm include the following steps:

1. Convert the data from the spatial domain into the frequency domain, the DCT formula is used as follows:
  (1) $$y_{i}=\omega_{i}\sum_{j=1}^{J}x_{j}\cos\left(\frac{\pi \left(j+0.5\right)i}{J}\right)$$
  where ω, *x*, *y*, *j*, *i*, *J* denote the scaling factor, the original data, the DCT vector, index of data *x*, the DCT vector index, and the length of the data *x*, respectively. The scaling factor is defined as $\omega_{i}=\frac{1}{\sqrt{J}}$ for i=1; otherwise $\omega_{i}=\sqrt{\frac{2}{J}}$.
2. Calculate energy concentration among the DCT vector to define the frequency threshold for distinguishing values. The DCT vectors *y* are sorted in descending order which is denoted as: $y=\{y_{n},\;y_{n-1},\;y_{n-2},\;\ldots ,\;y_{1}\}$. Define *i*, which determines how many frequencies that are required to represent the amount of the energy in the signal by using energy concentration threshold (*σ*), where 0 < *σ* < 1.
  (2) $$\frac{norm\left(y\left[1:i\right]\right)}{norm\left(y\left[1:N\right]\right)}<\sigma$$
  where the *norm*(·) is calculated by the Euclidean normalization (*p-norm*, *p* = 2) of *y*.
3. Apply an IDCT formula to reconstruct the data from the remaining DCT vectors, defined as follows:
  (3) $$x^{\prime }{}_{i}=\omega_{i}\sum_{j=1}^{J}y_{j}\cos\left(\frac{\pi \left(i+0.5\right)j}{J}\right)$$
  where ω, *y*, *x′*, *j*, *i*, *J* denote the scaling factor, the remaining DCT vector, the reconstructed data, index of the DCT vectors, index of the reconstructed data and the length of data *y*.

### 3.2. Design of Hardware Prototype

**Figure A1:**
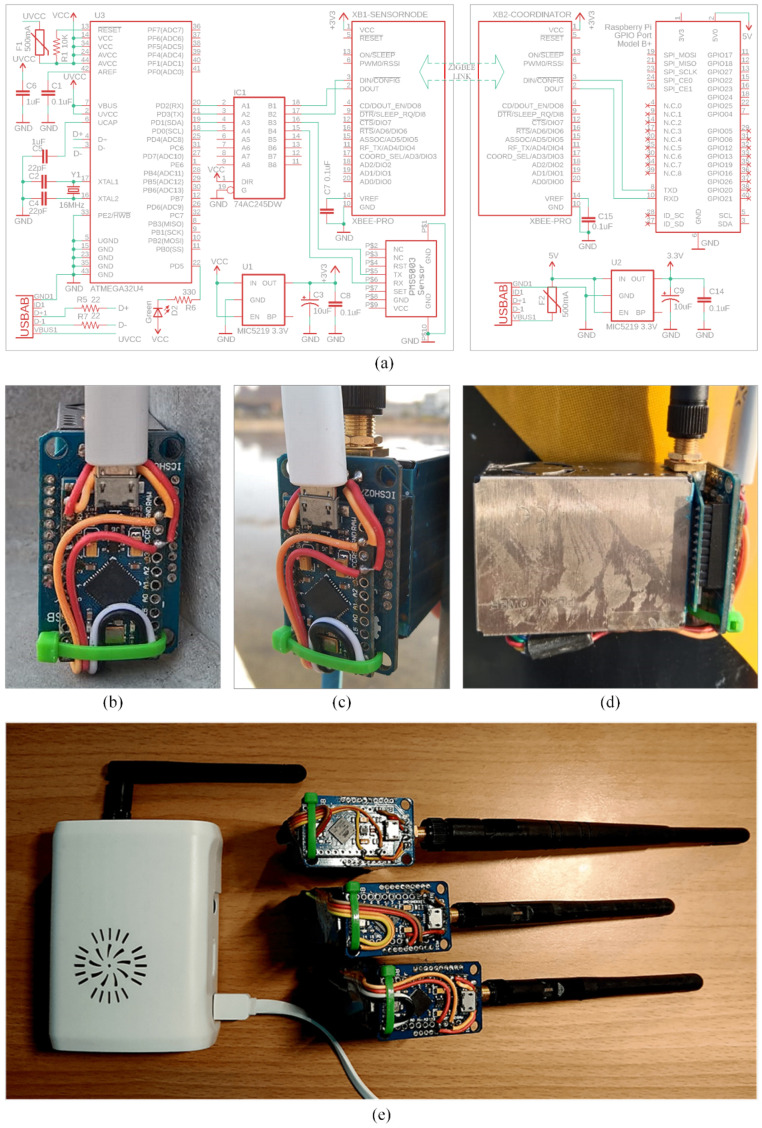
(**a**) The schematic of the prototype. (**b**–**d**) The deployment of the sensory nodes during real-time measurements. (**e**) The prototype that is used in data acquisition process involving a coordinator node (white) and three sensor nodes (blue). The sensory nodes use ATmega 32u microcontrollers while the coordinator nodes use Raspberry Pi 3 B+.

A small network consisting of four prototypes attached in Figure A1 includes of a coordinator node and three sensor nodes. There are three main parts that build the system architecture, i.e., sensor node, coordinator node, and aggregation server. The sensor node is developed using 8-bit microcontrollers AT-mega 32u with a clock speed of 16 Mhz. Each sensor node is equipped with a PMS5003 particulate matter sensor which is connected to a microcontroller via a serial interface. These sensors can measure several sizes of particulate matter (i.e., PM1.0, PM2.5, PM10), temperature, and humidity. Another serial interface is used to connect the microcontroller with XBee Pro S2C module. This module provides wireless connection capability (i.e., ZigBee network), to the microcontroller. To balance the voltage level between interfaces, an IC 74AC245DW is used as a level shifter. To convert the voltage to 3.3 V on the system, IC MIC5219 is applied. The sensor node is supplied by an external power supply from a 5 V battery. Each sensor node is capable of performing the DCT compression on a small sequence of data. These nodes also have the ability to enter sleep mode. The sleep mode is activated to save processing power leading to a decreasing in energy consumption. Meanwhile, the coordinator node is powered by Raspberry Pi 3 B+ and XBee Pro S2C module to collect the compressed data from the sensor nodes, then transmit it to the aggregation server. Finally, the coordinator uses Wi-Fi or Gigabit LAN port to connect to the server via an Internet router.

### 3.3. Software Development

**Figure A2:**
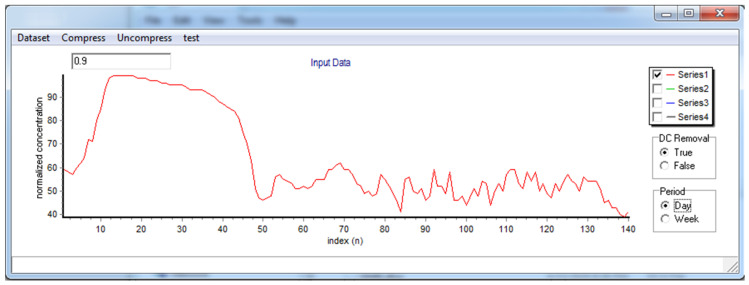
The PM2.5 prediction interface uses an LSTM model that is trained across decentralized coordinators.

The lossy compression technique is suitable to be applied to data generated by IoT sensors, especially related to weather monitoring, where the data is relatively and periodically stable with a low level of randomness. A data transmission framework is needed to maintain the data communication efficiently in WSN, described in Algorithm 1. The user interface to monitor the processes is also created and shown in Figure A2. A coordinator provides services for several sensor nodes in a ZigBee network. Furthermore, the data are transferred in a compressed format to provide efficient data transmission while reducing missing records and maintaining data integrity. 

First, the coordinator will send a message (*c*) in the ZigBee network to read data on one of the nodes. All sensor nodes via the interrupt mechanism will receive the message. Only the corresponding node gives the response back, meanwhile, the other nodes remain in sleep mode. The corresponding node will start the data recording process. A 30 s delay is required before the PMS sensor module provides stable measurement results. Measurements are made using sampling rate (*N*). After reaching *N* samples, i.e., specified by the coordinator, the sensor node converts the data *x* into *y* by using the DCT, compresses it, and then sends it to the coordinator via the ZigBee network. After the data *y* are sent, the sensor node enters the sleep mode again. Meanwhile, the coordinator receives the data *y* periodically from each node and uses it on an aggregate learning scheme, i.e., coordinated by an aggregation server. After that, all of the coordinator nodes send the learning model simultaneously to the aggregation server via the Internet. Finally, the prediction results $\hat{y}$ can be monitored from user interface that is provided by the server.
**Algorithm 1** Compressed Sensing**Input**: Command (*c*) **Output**: Compressed Data (*y*)**function** Compression () Serial.interrupt () **if** (Serial.read (*c*) == true)  sleep (false)               # activate the node  PMS5003.sleep (false)            # activate the sensor  *x* = PMS5003.read{*x*1, *x*2, *x*3, …, *x_N_*}     # read the sensor  *y* = dct(*x*)                   # compress the data  PMS5003.sleep (true)  sleep (true) **end if** **return**: *y*

## 4. Privacy-Preserving Prediction Model with Federated Learning

This section describes the concept of federated learning that preserves data privacy on a WSN network. This scheme is performed by coordinators and an aggregation server. The neural network model (i.e., long-short term memories) is constructed on every distributed compute unit (i.e., coordinator); meanwhile, the central server organizes the FL scheme that aggregates the learning model from the coordinators.

### 4.1. LSTM for PM 2.5 Prediction

**Figure 3:**
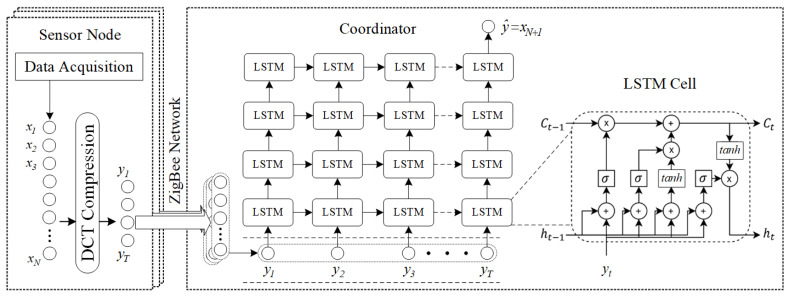
The proposed PM2.5 prediction system utilizes LSTM model on compressed datasets.

It has been shown that Long-Short Term Memories (LSTMs) can be used prediction systems to forecast sequence data [43,44]. In order to construct the proposed model, several layers of LSTMs are compiled as shown in Figure 3. In this model, the data $x_{N}$ are extracted from PMS5003 sensor with a data rate of *N*. Afterwards, the data are converted into compressed data $y_{t}$; then they are used as the LSTM input layer. The number of *N* varies up to 50 depending on the length of the compressed data, which can be smaller if the data can be compacted more densely. The LSTM uses two gates to control the content of the cell state *c*. One is the forget gate, which determines how much of the cell state $c_{t-1}$ at the previous time is retained to the current time $c_{t}$. The other is the input gate, which specifies the amount of network input $y_{t}$; at the current moment it is saved to the unit state $c_{t}$. The LSTM uses output gates to control how much of the unit state $c_{t}$, i.e., output to the current output value $h_{t}$. In each round of the LSTM network update, the cell accepts the hidden state $h_{t-1}$ and input $y_{t}$ of the previous cell in the cell sequence. The cell controls whether or not to discard a certain calculation process which defines the output. $f_{t}$ of the forgetting gate is shown in Equation (4), where *ζ* is the sigmoid activation function; $W_{f}$ is the forgetting gate weight matrix; $b_{f}$ is the forgetting gate bias term.

The basic idea of this LSTM network is to determine the retained information through a layer containing an activation function and then generate the cell state $c_{t}$ at the current t. The estimation process is considered as a combination of the following two calculations. Equation (5) calculates what information in the current cell input $y_{t}$ needs to be saved to the current state $c_{t}$ of the long-term and short-term memory network cells. The sigmoid layer of the input gate layer determines which information needs to be updated. In addition, in Equation (6), the *tanh* layer generates a vector which is the alternative content to update. Finally, the final output $\hat{y}$ of this LSTM network is the prediction of the next sensory value $x_{N+1}$.

(4) $$f_{t}=\zeta \left(W_{f}\cdot \left(h_{t-1},y_{t}\right)+b_{f}\right)$$

(5) $$i_{t}=\sigma \left(W_{i}\cdot \left(h_{t-1},y_{t}\right)+b_{i}\right)$$

(6) $${\tilde{c}}_{t}=\tanh\left(W_{c}\cdot \left(h_{t-1},y_{t}\right)+b_{c}\right)$$

### 4.2. Federated Learning for PM2.5 Prediction

**Figure 4:**
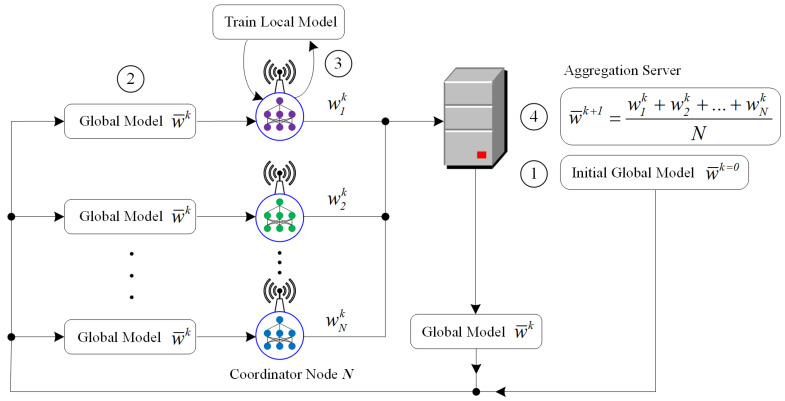
The FL scheme provides privacy preservation by training the dataset locally without exposing them directly over the network.

Federated compressed learning is based on compressed sensing and distributed learning, and its parameters updating method is similar to the basic idea of federated learning. In this paper, the federated compressed learning is focused on the top layer of the framework near the central server to train the prediction model. At first, the lower layer (i.e., near the edge) transmits the compressed data to the regional micro cloud or the fogs. The aggregation learning is provided by the coordinators and the aggregation server by updating global model regularly based on the local training models. FL architecture in its basic form consists of a curator or server that is coordinates training activities with the aggregate nodes (i.e., the coordinators). Clients are mostly fog devices that can reach thousands. These devices communicate at least twice with the server per training iteration. In Figure 4, the aggregation server initiates the global model (1). Then, each client receives the current global model weight from the server (2). Then, each client trains it on each of its local data (3) to generate updated parameters which are then uploaded back to the server for aggregation (4). This communication cycle continues until a predetermined number of periods, or an accuracy condition is reached. In this scheme, the aggregation is performed using the averaging operation. Algorithm 2 describes the pseudocode of the training process in this scheme.

The initialization process is started by randomizing the weights at *k* = 0 as a global model $w_{}^{k=0}$. Then, the global model is distributed to each coordinator. Each coordinator has collected compressed dataset from each sensor node in the lower layer of a local dataset $\left(y_{n},{\hat{y}}_{n}\right)$. By using this local dataset, each coordinator trains $\left(opt_{n}\right)$ the LSTM model using mean absolute error function (*MAE*) to generate a new local model $w_{n}^{k}$. In the end, the aggregation server generates and distributes a new global model ${\bar{w}}_{}^{k+1}$ by averaging all local models $w_{n}^{k};n=1,2,3,\ldots ,N$. Therefore, the communication on the central network does not involve data from the edge, but only collections of aggregated models which have no direct correlation with the dataset.
**Algorithm 2** Federated Learning for PM2.5 prediction with Federated Average (FedAvg)Input:
- Dataset $d_{i}=(y_{n},{\hat{y}}_{n})$
- *N* number of the client (NB-IoT Sensor Node Coordinator)
- Number of communication round *K*
- Initial Global Model $\bar{w}$

**Output**: Final Global Model**function** Federated_Learning ()
${\bar{w}}^{k=0}=\left\{w_{1},w_{2},\ldots ,w_{3}\right\}$ ← randomize parameters    # initialize global model (1)
**for** *k* = 1 to *K* **do**
  **for** *n* = 1 to *N*
**do**
   $w_{n}^{k}={\bar{w}}^{k}$                    # deploy global model (2)
   $w_{n}^{k}=opt_{n}\left(MAE\left(fw_{n}^{k}\left(y_{n},{\hat{y}}_{n}\right)\right)\right)$        # train local model (3)
  **end for**
  ${\bar{w}}_{}^{k}=\frac{1}{N}\sum_{n=1}^{N}w_{n}^{k}$                  # update global model (4) **end for** **return**: a global model ${\bar{w}}^{k}$


## 5. Experiments and Results

In this section, the datasets are analyzed statistically. Then, the compression parameter is tuned to achieve a lower data saving ratio with a lower error rate. By using the tuned parameter, a performance comparison for each FL scheme is provided. Finally, the security analysis is presented at the end of the section. In addition, the experimental results are performed by using prototypes with hardware specifications defined in Table 1.

### 5.1. Evaluation

The following metrics are used to evaluate the CS performance: data saving ratio and error rate. Meanwhile the FL performance is measured using mean absolute error.

1. Data saving ratio (*ς*): The smaller the data saving ratio, the more effective the compression algorithm is. The data saving ratio is performed by using the following equation:
  (7) $$\varsigma =\frac{\left\{X\right\}-\left\{Y\right\}}{\left\{X\right\}}$$
  where {*X*} is the size of original data *x* and {*Y*} is the reduced size of DCT vectors.
2. Error rate (*ε*): The smaller the error rate, the more effective the compression algorithm is. The error rate calculation performed to a set of reconstruction data, is given by:
  (8) $$\varepsilon =\frac{1}{I}\sum_{i=1}^{I}\frac{\left|y_{i}-{x^{\prime }}_{i}\right|}{{x^{\prime }}_{i}}$$
  where *x* is the original data, *x*′ is the reconstructed data, and *I* is the index of the data.
3. Mean absolute error (*MAE*): is a loss function on neural network model that can be applied to data with large outliers. This value is calculated as the average of the absolute difference between the actual and the predicted values. The model can be updated to use the *MAE* loss function. The *MAE* performed by each FL variants, is given by:
  (9) $$MAE=\frac{1}{I}\sum_{i=1}^{I}\left|{\hat{y}}_{target\left(i\right)}-{\hat{y}}_{predicted\left(i\right)}\right|$$
  where ${\hat{y}}_{target\left(i\right)}$ is the target data, ${\hat{y}}_{predicted\left(i\right)}$ is the prediction data, and *i* is the index of the data.

### 5.2. Dataset Characteristic

The compressed sensing algorithm is evaluated by using datasets that were recorded during September 2020. The datasets consist of a large dataset obtained from the Airbox system, which includes 1000 devices that spread across Taiwan and a small dataset, collected from the prototype. For this experiment, five variables (i.e., generated by PMS5003 sensor module) are measured from the prototype in a suburban district, Wufeng in Taiwan, i.e., consisting of PM1.0, PM2.5, PM10, temperature, and humidity. The dataset is collected with a time sampling of 5 min. The shorter the sampling time, the more the data that will be generated. Moreover, statistical assessments are used to evaluate the characteristics for each type of dataset. 

Table 2 presents statistical properties consisting of standard deviation (SD), normalized standard deviation (NSD), skewness, and kurtosis from the open dataset. The NSD is the statistical assessment that is used to determine how the data is scattered in the sample and how close the individual data points are to the mean value. Skewness is a value that shows the asymmetry degree of a dataset distribution. Skewness over 1 and below −1 indicates that the data is highly skewed. Meanwhile, kurtosis is the level of distortion in a distribution. A kurtosis i.e., higher than 3 or lower than −3 in a set of data, indicates that the data have heavy outliers. If a dataset has a high kurtosis value, an investigation is needed to find the cause of these many outliers. This might indicate an incorrect data entry or data sparsity because of the system’s weaknesses. 

In the datasets that are collected from the Airbox system, different statistical characteristics are observed for all metrics, and it can be divided into two groups, namely the PM datasets and the non-PM datasets. The PM1, PM2.5, and PM10 datasets have a much higher skewness value compared to the other datasets. For those three datasets, the kurtosis highly deviates from 3 when compared with the other two datasets. The distribution of those three datasets has values that change more sharply than those of the normal distribution. However, the kurtosis for temperature and humidity datasets is lower than 3, which indicates that the data distribution is closer to normal distribution. It can be concluded that the PM datasets obtained from the Airbox sensor network are distorted and fragmented. On the other hand, in the dataset that is recorded by using the prototypes, similar statistical characteristics are detected for all metrics. The prototypes are able to collect data with skewness and kurtosis value under 1 and 3 respectively. The data distribution in the datasets is not too deviated. The system is made only on a small scale. Thus, it minimizes the occurrence of fragmented sparse data. The larger the network, the more potential for sparse data to appear. Therefore, the weaknesses of complex and massive WSN are minimized by implementing the FCL method.

### 5.3. Tuning Energy Concentration Threshold

**Figure 5:**
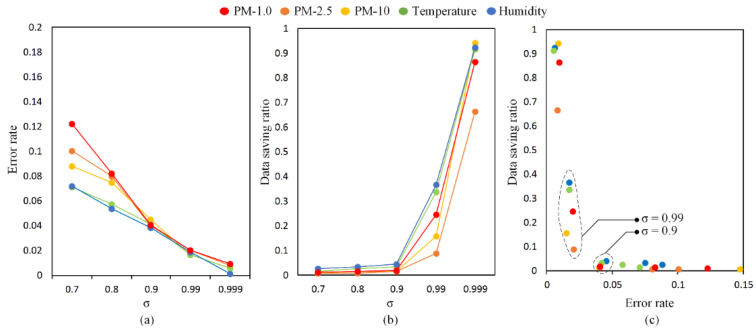
Comparison between (**a**) error rate and (**b**) data saving ratio with various *σ* on the reconstructed data. Meanwhile (**c**) evaluates the optimal value of *σ* that achieves efficient data generation while maintaining data fidelity.

Energy concentration threshold (*σ*) determines a series of signal properties that have a significant impact to redefine the signal. The energy concentration is calculated from its DCT vectors using Equation (2). From its energy concentration series, several threshold values are chosen: 0.7, 0.8, 0.9, 0.99, and 0.999. The data with energy concentration below the threshold represents a value that has no significant impact and can be ignored. This value is removed so that the DCT vector series will be drastically reduced. From the remaining DCT vector values, the signal is reconstructed. To evaluate the data fidelity, the original data is compared with the reconstructed data. The smaller the errors, the more efficient the compression algorithm. As shown in Figure 5a, from the five variables tested, the error rates look similar. The differences are not too far and look almost the same when the higher energy concentration threshold is used. The error rate starts to decrease below 5% when the energy threshold is set to greater than 0.9 for all data variables. 

In the next experiment, the data saving ratio is evaluated by changing the energy concentration threshold, shown in Figure 5b. The data saving ratio represents the comparison between the amount of compressed data and the amount of original data. The smaller the ratio, the better the compression algorithm used. This means that the original data can be compacted into smaller series of data. In other words, the storage capacity which can be saved is even greater. By changing the energy concentration threshold to its savings ratio, it produces linear results. The lower the energy concentration threshold, the smaller the data saving ratio. Moreover, by using the maximum data saving ratio, i.e., 0.999, the DCT could save storage a little bit. Furthermore, it is necessary to observe the effect of the data storage ratio on the error rate. The best parameters for the five variables should be observed to provide the most optimal compression results. The results are optimal if the data storage ratio is low and followed by a low error rate. In Figure 5c, the variation of data results for several different energy compaction thresholds is displayed. The optimal value is obtained for each variable is *σ* = 0.9, which achieves a low data saving ratio of around 95%. Meanwhile, by using a higher energy concentration threshold, (e.g., 0.99) the error rate decreases more but it sacrifices the data saving ratio. It means the amount of data that could be saved is not too much. Finally, this compressed data with *σ* = 0.9 will be used in further experiments related to the FCL scheme. As a comparison, another CS technique, i.e., discrete wavelet transform (DWT) is used to evaluate the efficiency of CS performance.

### 5.4. FCL Performance

**Figure 6:**
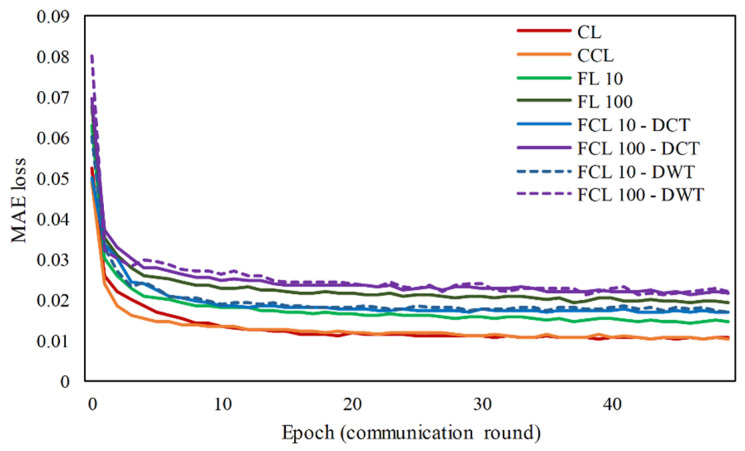
The *MAE* loss for eight different training schemes during 50 communication rounds.

From Figure 6, first, when the number of aggregate nodes is compared, even if the size of the epoch increases, it has an effect on reducing the reconstruction errors, i.e., evaluated by using *MAE* loss. Therefore, in the FL scheme, defining a number of nodes is an important key that affects the accuracy of the prediction system. Then, the prediction errors of the several algorithms are compared, i.e., Centralized Learning (CL), Centralized Compressed Learning (CCL), federated learning with 10 and 100 aggregate nodes (FL 10, FL 100), and federated compressed learning with 10 and 100 aggregate nodes with DCT-based and DWT-based algorithm (FCL 10–DCT, FCL 100–DCT, FCL 10–DWT, FCL 100–DWT). The overall *MAE* loss after 50 epochs for each algorithm is 2.05, 0.61, 1.97, 0.49, 6.54, 2.83, 6.36, 2.79, 6.39, and 2.78, respectively. Compared with the CL algorithm as a baseline, the *MAE* loss of FL 10 and FL 100 algorithm is increased by 29.8% and 65.7%, respectively. The more nodes involved in the FL scheme, the higher the *MAE* loss given by the training algorithm. In general, there is no significant difference between FCL using the DCT or DWT technique in term of training performance. However, the DCT variant generates a more compact data than the DWT variant. Meanwhile, when comparing the *MAE* loss of CL scheme with those of FCL 10 and FCL 100, the training losses increase by around 100% and 120%, respectively. Thus, even under different network configurations, the FCL algorithm increases prediction errors and decrease data reconstruction accuracy. However, the decline in training performance has not shown the general performance of the prediction system. These results are only a raw description of the declining training loss of the FCL scheme compared with the centralized training.

**Figure 7:**
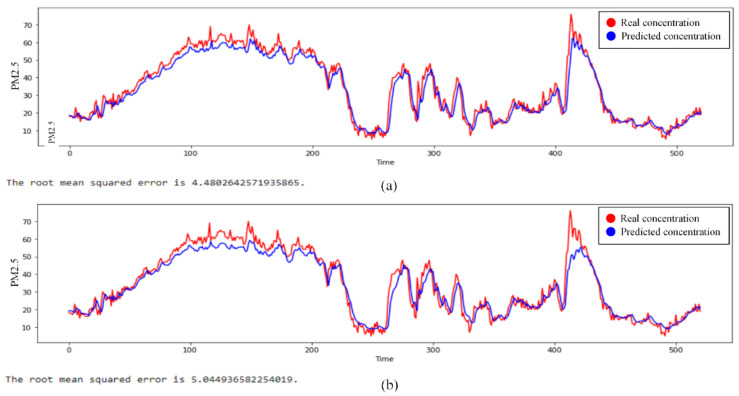
The PM2.5 prediction result utilizing (**a**) CL compared with (**b**) FCL 100–DCT using the global model that is trained in 50 epochs.

For a deep understanding of system performance in predicting the PM2.5 concentrations, an inference procedure is performed on the dataset by using the best performance (i.e., CL) and the worst performance (i.e., FCL) achieved by the training scheme based on Figure 6. Finally, the prediction performances are shown in Figure 7. In general, all of the FL-based schemes produce a slight reduction compared to the results achieved using the CL scheme. Qualitatively, the prediction results still show good results even though the accuracy decreases. It can be seen that the data prediction resembles the ground truth. This is understandable because the difference in *MAE* loss is not too big, only in the range of 0.01. The results show a slightly decreasing 0.564 in the RMSE between both of them. However, the FCL scheme still has benefits in terms of data privacy. 

### 5.5. Security Analysis

The privacy protection capabilities of the proposed framework are described from the following standpoints. From the data access standpoint, the proposed framework, i.e., developed based on the FL, can protect the original data by only sending the prediction model parameters through the network. The core idea is to distribute the training mechanism to the edge networks performed by the smaller nodes. As long as the original data from the end nodes are not exposed to the network, the chances of being eavesdropped on can be minimized. Specifically, the federated model achieves a slightly lower accurate prediction of PM2.5 concentration compared with the centralized one; however, it guarantees information-based data privacy. From the data generation standpoint, the experimental results show that the performance of the proposed framework (i.e., the FCL) is comparable to the centralized model (i.e., the CL). The CL scheme needs to aggregate a massive amount of original data to achieve high-precision prediction of PM2.5 concentrations. Meanwhile, the FCL only collects compressed data from a smaller edge network and aggregates the parameters (i.e., not contain the original data) to the server. The FCL performs comparable results to a CL approach under the constraint of privacy preservation and it provides drastically data reduction over the conventional ones. The FCL also reduces the number of neurons that are needed to build the prediction model, which incurs a remarkable decrease in the local training time as well as the aggregated training time. Moreover, the chance of sparse data appear because of system complexity, can be reduced by using the denoising feature on the DCT technique. 

## 6. Discussion

**Figure 8:**
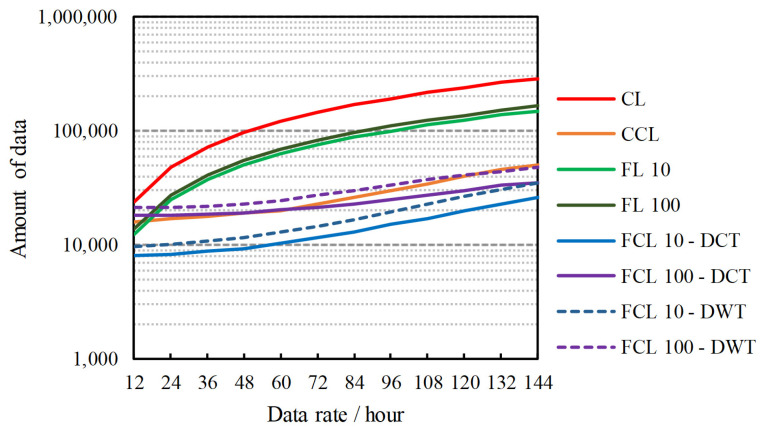
Simulation of data generation on WSN that utilizes different schemes with varying data rates.

With the increasing number of variables being measured, a massive centralized WSN is not suitable for use in this case. An approach is needed to transfer a massive amount of data efficiently while maintaining data privacy. As shown in Figure 8, the proposed scheme is capable of transferring and reconstructing data with a small amount of data. The differences between the reconstructed and the original data by using FCL were not very significant compared with that of using CL. In order to develop a federated learning scheme to adapt to ever-changing situations, it is necessary to conduct model training on a wider network over a longer time span. With the training that covers a broader network, the spatial features are better captured. Meanwhile, with the training on a longer dataset, (e.g., a year) temporal features could be better extracted. However, the larger and the longer the dataset, the more the data, the layers, and the parameters. This will increase the training period and increase transmission costs. Therefore, an edge-computing architecture is a solution. It means that every end device is equipped with a compute module that is capable to process a small-scale FL scheme. Furthermore, the training process is carried out in several stages, starting from the nodes near the edge, leading to FL in fog, until reaching the cloud. Finally, this scheme effectively avoids network problems such as data overload and inefficient training by utilizing a more compact and a more robust dataset.

A set of data sequences, i.e., collected from 1000 nodes in the Airbox system, is used to simulate the amount of data generation on more complex systems. This simulation calculates the amount of data that is generated for each scheme. The data generation is calculated by supposing that the maximum sampling period in an hour is up to 144 data. During this period, a sequence of data is recorded by each sensor node and each of them will contribute to the increasing number of data that is generated in the WSN. The greater the amount of data, the greater the chance that sparse data will appear, and the longer the time that is required to complete the process. This simulation is carried out by calculating the amount of data that is generated at the edge of the network (e.g., from the sensor node) and the number of parameters produced during aggregated training. The results are shown in Figure 8, where the greater the data rate used, the more data will be produced.

A slightly different pattern is generated by all schemes that use compressed sensing techniques. There is a significant reduction in the amount of data when using the CCL and FCL schemes. The difference becomes greater when using a larger data rate. This is in accordance with the results in previous tests where the CS method was able to provide a compression ratio of up to 95%. It is interesting to note that the DCT-based FCL scheme provides more compact data generation than the DWT-based FCL scheme. As described in [25,26], the DCT performs a higher compression rate rather than other techniques but, sacrifices a slight error rate. However, when faced with federated learning problems, both CS techniques (i.e., DCT and DWT) produce nearly identical learning features. These similar features are generated by the neural network, which simply translates the input into vectors that correspond to the targets. A small differentiation of the input vectors will not significantly affect the training performance. In addition, the FCL will greatly reduce computation time as well as network traffic. This scheme uses the CS method to compress data and the FL scheme to provide efficient federation training at the expense of a slight decrease in the accuracy of the prediction system. The proposed scheme has the potential to reduce data traffic and power consumption in WSNs, especially in a massive scale smart city sensing. In summary, Figure 8 shows that:

(1) WSN data generation is significantly compacted by the CS in comparison to conventional approaches without the CS. The efficiency of the CS in reducing the data is the key factor in the computation efficiency of the FCL.
(2) Data privacy is guaranteed by the FCL scheme at the top layer, while security at the bottom layer is maintained by the CS techniques.

The analysis process needs to be carried out carefully by identifying and positioning the performance metrics and trade-offs in relation to each other before concluding generalizations. The target is therefore to generate a cost and trade-off model that takes into account the following indicators, i.e., *MAE* loss vs. communication round, data generation vs. sampling resolution, and execution time of training vs. inference. Based on the experiments, the FCL generates a slightly higher error rate (i.e., measured with *MAE*) than the other schemes. However, as proof of concept, this scheme offers more efficient data generation and privacy preserving. Moreover, the proposed scheme produces an efficient amount of data with increasing number of data sampling resolutions. Theoretically, the greater the data processed, the more time required by the system to complete the training. The simulation notes that the execution time per epoch at the training stage for the CL, CCL, FL 10, FL 100, FCL 10–DCT, FCL 100–DCT, FCL 10–DWT, FCL 100–DWT scheme is 13 s, 3 s, 285 s, 2460 s, 207 s, 1845 s, 245 s, and 1985 s, respectively. These execution periods are correlated to the number of input dataset that have been compressed. Generally, all of the FCL schemes generate a longer execution time than the centralized scheme, but shorter than the conventional FL schemes. In a real-time application, only the inference engine contributes to the processing speed while generating prediction results. The execution time depends on hardware variations, the number of nodes, and communication delay. However, the processing speed of the inference engine will not differ too much for all schemes because basically, the inference engine only processes one-time forward propagation in neural networks. Finally, other practical considerations regarding the implementation feasibility of these machine-learning models are parallelization over multiple cores or nodes and the availability of hardware-accelerated infrastructure.

## 7. Conclusions

In this paper, the FCL framework in which the core idea inherits compressed sensing combined with federated learning is proposed to reduce data sparsity. Based on that, an edge-computing architecture to develop an inference framework for smart cities sensing is provided. This architecture could easily and simultaneously solve the problems of node deployment, communication efficiency, and fragmented records while preserving data privacy. The proposed framework could be implemented by using the WSN nodes and built from a small low-powered compute unit, i.e., an IoT application suitable for a smart city. This study also extends the FL scheme to the FCL scheme for broader application scenarios with thousands of the end devices. In the future, the multi-layer LSTM in the FCL scheme will be developed and used to solve the distributed prediction problem in more application fields.

## Figures and Tables

**Table 1:** The specifications of the prototypes used in the experiments.

Properties	Sensor Node	Coordinator Node	Aggregation Server
Processor	Single Core ATmega 32u	Quad Core Broadcom BCM2837B0	Dual 20-Core Intel Xeon E5-2698 v4
Architecture	8-bit AVR	ARMv8	x86-64
Clock speed	16 MHz	1.4 GHz	2.2 GHz
RAM	2.56 KB	1 GB	256 GB
Network interface	Serial XBeePRO S2C	Serial XBeePRO S2C Wi-Fi 2.4 GHz Gigabit LAN	10 Gigabit LAN
Storage	EEPROM 1 KB	MicroSD 32 GB	SSD 1.92 TB
PM Sensor	Plantower PMS5003	-	-
Power consumption (*watt*)	0.9	3.6	1600

**Table 2:** Statistical characteristics of dataset that is used for experiments.

Dataset	Variable	SD	NSD	Skewness	Kurtosis
Airbox (1000 nodes 78.5 MB)	PM1.0	12.315	0.018	3.173	78.599
	PM2.5	17.513	0.022	3.184	76.301
	PM10	22.947	0.021	1.969	42.091
	Temperature	3.401	0.074	0.142	0.853
	Humidity	13.192	0.167	0.117	−0.374
CS Prototype (4 nodes 437 KB)	PM1.0	1.949	0.452	0.897	0.546
	PM2.5	1.845	0.223	0.692	0.980
	PM10	2.345	0.167	0.622	0.977
	Temperature	6.385	0.336	0.210	0.649
	Humidity	17.060	0.247	1.044	0.336

## Data Availability

Data sharing not applicable.
